# Beyond ad hoc home blood pressure monitoring: The need for standardisation and integration into primary care

**DOI:** 10.1038/s41371-026-01202-0

**Published:** 2026-08-19

**Authors:** Annika Wilson, Suzanne Waddingham, Laura Sutton, Rishita Chandra, Belinda Donovan, Mark R. Nelson, James E. Sharman

**Affiliations:** 1 Menzies Institute for Medical Research, University of Tasmania, Hobart, TAS Australia; 2 Tasmanian School of Medicine, University of Tasmania, Hobart, TAS Australia; 3 Consumer Representative, Devonport, TAS Australia

**Keywords:** Hypertension, Health care

## Abstract

Hypertension guidelines recommend home blood pressure monitoring (HBPM) using a standardised protocol to ensure accuracy and guide effective management. However, the extent to which HBPM is performed according to standardised protocol is unclear. The study aimed to explore how HBPM is used and understood by both health professionals and community members. A targeted, qualitative content analysis was conducted using a hybrid approach that combined deductive and inductive strategies to identify themes related to HBPM. Data was collected across 17 consultations and 19 co-design workshops with community members (adults 18 years and above, *n* = 154), and primary health professionals (general practitioners, general practice staff, nurses, health workers, pharmacists, *n* = 73) for a total of 227 participants. Sessions included unprompted discussions about experiences with HBPM, perceptions of accuracy, and practices in BP measurement and were conducted across three Tasmanian regions. Both community members and health professionals reported HBPM to be valuable and practical for hypertension management. Community members consistently ranked HBPM as a key preference for where they wanted their BP measured. However, there was variability in HBPM technique and limited awareness of evidence-based protocols. Instead, participants described ad hoc, individualised, and often invalid methods for measuring and recording BP at home. HBPM is an acceptable and widely used strategy for monitoring hypertension, but ad hoc, non-standardised use in practice undermines its accuracy and clinical utility. Addressing barriers to standardisation and integration into primary care are essential for optimising the role of HBPM in hypertension management.

## Introduction

Hypertension is a major cause of preventable cardiovascular diseases and a leading contributor to premature mortality and disability worldwide [1, 2]. Nearly one in three Australian adults is affected, yet hypertension detection, treatment, and control rates remain suboptimal, particularly in rural and regional areas where access to primary care is limited [3–5]. Accurate and consistent blood pressure (BP) measurement underpins the diagnosis and management of hypertension [6]. In this context, home BP monitoring (HBPM) is an important adjunct to clinic-based and ambulatory BP readings, offering the potential for more representative measurements, earlier detection of changes in BP, and greater patient engagement in self-management [7]. Importantly, BP values obtained through HBPM have been shown to be more strongly associated with cardiovascular morbidity and mortality than clinic BP measurements, underscoring the clinical utility of HBPM for cardiovascular risk prediction and management [8].

HBPM involves individuals taking their own BP readings outside of clinical settings, over multiple days, and sharing results with their healthcare provider. When performed according to standardised protocols, it can improve diagnostic accuracy, reduce the influence of white-coat and masked hypertension, and support informed treatment decisions [9, 10]. These benefits are well recognised in current Australian and international hypertension guidelines [7, 9, 11–13], which recommend HBPM as part of routine hypertension management.

However, the accuracy and clinical value of HBPM depends heavily on its correct use. Evidence shows that both patients and health professionals often lack awareness of standard protocols, leading to inconsistent technique, variable recording, and misinterpretation of results [10, 14]. Common issues include not using devices that have been properly tested for accuracy (validated) [15], incorrect cuff placement, insufficient rest time, and selective reporting of preferential readings. Such deviations can undermine the reliability of HBPM data, erode clinician confidence [14], and potentially delay or misdirect treatment.

Despite growing interest in HBPM [16], little is known about how it is currently understood, used, and integrated into everyday community and primary care contexts. Previous research has largely focused on clinical trials or program evaluations, with limited attention to real-world experiences and practices of those using HBPM regularly [17–19]. Gaining insight into these perspectives, including the barriers and enablers to consistent use, is essential to promoting guideline-aligned HBPM and realising its full potential in hypertension management. This study explored discussions about the practices, experiences, and perceptions of HBPM among community members and primary health professionals that came from a larger BP study.

## Methods

### Study overview

This paper reports findings from a larger program of research designed to identify and address local priorities for improving hypertension prevention and management in Tasmania. Data was collected from 17 consultations and 19 co-design workshops with community members and primary health professionals across 14 towns in three Tasmanian regions. Consultations were semi-structured, facilitated group discussions and stakeholder meetings with community members, health professionals, and community organisation representatives, to explore local experiences of cardiovascular health and hypertension care, perceived gaps, and priorities for improvement. Co-design workshops were structured, interactive sessions that built on these discussions to collaboratively refine problems and co-develop strategies to improve hypertension prevention and management.

All sessions were conducted using a Design Thinking methodology, a human-centred and collaborative approach that draws on empathy for community needs and applies co-design principles to generate solutions with communities, rather than for them [20].

During these discussions, the topic of HBPM emerged without prompting across both health professionals and community members. Given the frequency and salience of these discussions, a targeted, secondary analysis was undertaken to explore HBPM-specific experiences, perceptions, and practices in greater depth for this paper.

Ethics approval was granted by the University of Tasmania Human Research Ethics Committee (project ID 28339). All participants provided written informed consent. This study is reported in accordance with the Standards for Reporting Qualitative Research checklist [21].

### Participant selection and recruitment

Health professionals included general practitioners (GPs), general practice nurses and other staff, pharmacists, and allied health workers who routinely measure BP in primary care. Community participants were adults ≥ 18 years with lived and living experience of hypertension and/or BP measurement in primary care.

Purposive and snowball sampling in the larger program of research ensured representation from rural and regional areas across Tasmania’s three main regions (Northwest, North, and South). Sampling saturation was determined through both data saturation and geographical coverage with a focus on the Northwest due to existing health inequities and unique community needs [3–5].

### Consultation and workshop procedures

Data collection occurred across two phases between May–September 2023 and July–September 2024. Sessions lasted 60–90 min and were scheduled at times and locations convenient for participants, with sessions delivered either in community venues or via video conference. A standardised PowerPoint presentation ensured consistency across sessions and provided background information on hypertension, the broader project aims, local health service context, and (where available) emerging findings from previous consultations. Discussions were guided by semi-structured prompts exploring participants’ understanding of hypertension, how they knew whether they had high BP, where they sought information and support, barriers and facilitators to hypertension prevention and management, and opportunities to improve local services and community support. Example questions included: *“What comes to mind when you hear the term high BP?”*, *“How do you know if you have high BP?”*, and *“Where do you go for information or advice about high BP?”*. All participants completed a short REDCap survey capturing key demographic information (age, gender, and employment status).

Sessions began with open discussion of BP measurement experiences, guided by semi-structured prompts about BP monitoring use, barriers, and supports. Although clinic-based BP measurement was the intended focus, participants often initiated discussions about HBPM, covering device use, perceived accuracy, and monitoring routines.

In larger sessions (i.e. ≥ 5 participants), participants broke into smaller focus groups to identify priorities for improving BP measurement. Each group listed their priorities, which were then ranked using “dotmocracy” voting with adhesive dots. As part of this broader priority-setting activity, community members also ranked preferred locations for BP measurement. Health professionals did not participate in this activity. Detailed methods and results from the broader co-design priority-setting exercise have been reported elsewhere [22]. Discussions were audio-recorded and transcribed verbatim via automatic transcription or third-party service. Transcripts were proofread, de-identified, and labelled by workshop number and participant type (e.g., Community Member [CM] 1; General Practitioner [GP] 2; and Other Health Professional [HP] 3). All data was stored in a secure folder.

### Data analysis

Demographic data were summarised using descriptive statistics. Priority-ranking data from dotmocracy were collated in Excel spreadsheet and analysed using summative content analysis [23]. Qualitative content analysis was conducted using a hybrid approach that combined deductive and inductive strategies in NVivo version 20.0 (QSR International). Initially, a directed content analysis was applied to identify and code targeted keywords relating to HBPM. This deductive phase ensured that predefined concepts were systematically captured across the dataset. Following this, an inductive analysis was undertaken to explore patterns and themes emerging beyond the initial coding frame. Coding and interpretation were conducted independently by two researchers (AW/SW) with discrepancies resolved through discussion with a third researcher (JS). These themes were refined iteratively, with attention to identifying similarities and differences between health professionals and community perspectives. Triangulation was achieved by comparing themes with priority-ranking data and notes from the workshops to strengthen credibility.

### Researcher characteristics and reflexivity

The broader research team included expertise in cardiovascular, primary healthcare, and community-based participatory research. Sessions were facilitated by SW (experienced in consumer and community involvement and participatory and community-based mixed methods research approaches) and co-facilitated by LS (public health researcher with experience in mixed-methods research) and JS (BP measurement expert). While JS’s knowledge could have influenced workshop discussions, reflexive strategies, such as using semi-structured prompts, were employed to mitigate bias. Three researchers (SW/LS/AW) were involved in the analysis with experience in qualitative research methods.

## Results

### Participant characteristics

227 individuals participated in the study. Most were community members (*n* = 154; 67.8%), with the remainder primary health professionals (*n* = 73; 32.1%). Women comprised 72.2% of participants (*n* = 164). Community members had a mean age of 54.3 years ( ± 17.9). The majority of participants resided in Northwest Tasmania (93.8%). Among those reporting employment (*n* = 224), almost all health professionals were in paid employment (98.6%), compared with 35.7% of community participants. Health professionals represented diverse roles, including GPs (47.9%), GP clinic staff (20.5%), nursing staff (13.7%), pharmacists/pharmacy staff (9.6%), and other primary care roles (8.2%).

These findings reflect a diversity of experiences, motivations, and practices related to HBPM. While overlapping perspectives were evident, health professionals primarily conceptualised HBPM through a clinical lens, focusing on accuracy, feasibility within practice workflows, and patient adherence to standardised BP measurement protocols. However, many also described ad hoc approaches to HBPM characterised by inconsistent guidance, informal follow-up, and variable integration into routine care. In contrast, community members often viewed HBPM as a personal health management tool, emphasising its value in everyday life, accessibility of equipment, and the reassurance it provided outside clinical encounters. Yet, their experiences similarly reflected ad hoc practices, including irregular monitoring routines, limited feedback from clinicians, and uncertainty about correct technique or interpretation of results.

Four overarching themes were identified: (1) HBPM as a strategy for self-monitoring, prevention, and personalised care; (2) Variability in HBPM technique and limited awareness of evidence-based protocols; (3) Balancing comfort and convenience with accuracy; and (4) Trust, connection, and follow-up are critical to sustained use of HBPM. Verbatim quotes are provided to illustrate key findings.

### HBPM as a strategy for self-monitoring, prevention, and personalised care

Participants described a variety of reasons for undertaking HBPM, often linked to personal health concerns, self-preservation, and ongoing clinical management. For both community and health professionals, HBPM was driven by a desire to observe trends over time, particularly in relation to medication adjustments, rather than focusing on individual readings. One participant explained, *“I can say just an example, because my GP got me to do this, if I go back to September, October last year and see what the readings are and then once medications were put on, it was tracking it. [My GP’s] quite happy to look at that because he’s not looking at the numbers, he’s looking at the trends.”* – CM 1.

Others expressed strong personal motivations, framing HBPM as a preventative tool to manage risk and avoid adverse health events. For example: *“If I know my BP is high, I take my BP regularly at home because I want to make sure that it’s not going to kill me.”* – CM 2. For some, HBPM also offered a way to overcome the “white-coat effect”, where BP readings are artificially elevated in clinical settings due to anxiety. One participant described monitoring at home to obtain readings they considered more representative of their usual BP: *“I found I’ll go to the doctor and… with that white coat, my BP will be through the roof. Then I’ll monitor it at home over the next couple of weeks and it will be spot on.”* – CM 3.

### Variability in HBPM technique and limited awareness of evidence-based protocols

Overall, there was substantial variation and ad hoc approach to how participants conducted HBPM, with many reporting limited awareness of guideline-recommended protocols. Most relied on self-developed routines, online searches, or device instructions, with few describing comprehensive adherence to evidence-based practices. When asked if they had received guidance by their medical provider, a community member responded, *“[Nobody’s told you?]. No. No, just the instructions that came with the machine, a bit of basic Google searching.”* – CM 4.

Some participants shared examples of inaccurate or inconsistent measurement practices, including selective recording of readings or reliance on non-validated devices. One GP noted the risk of inaccurate reporting: *“There’s some really great research where people have faithfully written down in the diary the numbers, not realising that the BP machine was storing all the information, they hadn’t been told that… they’re not always the same for various reasons.”* – GP 1.

Measurement routines were often shaped by personal comfort, perceived urgency, or concern over specific readings. One participant described repeating measurements multiple times in quick succession, particularly when faced with unexpectedly high results: *“I do three blocks... and then I leave it, and then I’ll go and do whatever I’ve got to do, ra, ra, ra, come back, sit down in the chair, wait 10 min or so, do three blocks again… if I’m really, really worried, like if it’s around the 230-240, I’ll sit there for another half an hour, then do three blocks again.”* – CM 5.

### Balancing comfort and convenience with accuracy

Participants described several barriers and enablers to HBPM. In general, HBPM was perceived as more comfortable and potentially more accurate than clinic-based measurements, largely due to the ability to rest and prepare in one’s own environment. As one community member noted: *“For the accurate reading that you’re talking about, needing to be calm and rested, you’re only going to get that at home. You’re not going to get that out and about. People are naturally uncomfortable.”* – CM 6.

However, some participants also highlighted practical barriers, including lack of access to validated equipment, insufficient knowledge of correct technique, and the anxiety associated with frequent monitoring. The need for clear education was emphasised by health professionals: *“It’s an issue, because you can give people the instructions and you can hope and trust them [that] they do what the instructions say.”* – GP 2.

A community member described feeling unsettled by the unpredictable nature of their HBP readings, noting that moment-to-moment fluctuations could be confusing. These variations, sometimes accompanied by physical sensations such as dizziness, led them to question the reliability of their results and to monitor repeatedly in search of reassurance. As they explained, *“Sometimes it [the result] just rocks you and you get giddy, and you go and test it and it’s too low, and then all of a sudden it shoots up in the next five min.”* – CM 5.

### Trust, connection, and follow-up are critical to sustained used of HBPM

Trust in both the measurement process and the individuals interpreting the results emerged as an important factor influencing HBPM engagement. GP endorsement and integration into ongoing care increased health professionals’ confidence in HBPM. For example, *“They would be more likely to have it done this way too if the GPs were on board. So, if the GPs are on board, they trust it.”* – HP 1.

Social connection was also a motivator, particularly for individuals experiencing isolation within the community. *“They have no one that comes all week and they know I do home visits and do their BP, so they invite me to come and do their blood pressure because they’re lonely a lot of time.”* – HP 1.

Additionally, participants highlighted the importance of follow-up systems to support the use of HBPM. Discussions suggested that, while HBPM was viewed positively, there was less clarity regarding how readings should be communicated and incorporated into ongoing care. As one participant queried, *“Would you [community members] like to be contacted by a doctor to have a discussion about your results? Would you like the results to be sent through to your GP so your GP contacts you?”* – GP 4. While participants generally responded positively to these suggestions, the discussion did not progress beyond brief affirmations. Nevertheless, the exchange highlighted broader considerations regarding how HBPM results could be communicated, reviewed, and linked back into routine hypertension care.

During open-ended discussions, community members described having their BP measured across a range of settings, including general practices, pharmacies, community settings, and at home. As part of a separate priority-setting dotmocracy activity, participants also ranked preferred locations for BP measurement. Home BP measurement emerged as one of the preferred options identified by participants.

## Discussion

This study provides new insights into how HBPM is understood and used by both health professionals and community members. Consistent with international literature, our findings show that HBPM is valued for its role in tracking BP trends, identifying cardiovascular risk, informing treatment, and reducing the influence of white-coat hypertension [8, 24]. Similar to recent Australian and international evidence, participants did not consistently follow guideline-recommended protocols [14, 25]. Instead, HBPM was often performed in ad hoc and individualised ways, with limited awareness of evidence-based technique and inconsistent integration into routine care.

A key implication of this study lies in the differing framings of HBPM between health professionals and community members. Health professionals tended to view HBPM through a clinical lens, emphasising accuracy, workflow feasibility, and adherence to protocols, reflecting concerns commonly reported in guideline and implementation literature [7, 9, 26]. In contrast, community members situated HBPM within the context of daily life, valuing it for accessibility, reassurance, and integration into self-care practices, echoing findings from qualitative studies internationally [16, 19]. This divergence underscores HBPM’s dual function both as a clinical instrument to guide diagnosis and treatment, and as a self-management strategy.

Barriers identified in this study, such as lack of access to validated devices, reliance on device instructions or online searches, and limited feedback pathways, mirror international reports of low-quality HBPM and poor clinician confidence in patient-reported readings [27, 28]. Without systematic support, variability in technique undermines the reliability of HBPM and reduces its clinical utility. Importantly, participants in our study expressed strong motivation to measure BP at home, suggesting that with the right education, equipment, and feedback systems, high-quality HBPM is possible.

These findings reinforce the importance of standardised HBPM education for both patients and health professionals. Evidence from previous studies shows that education interventions can improve adherence to recommended HBPM protocols for both health professionals and patients [29], particularly when designed to meet health literacy needs and delivered in structured formats [30, 31]. In our study, participants who sought guidance from health professionals, often received only brief or vague instructions, which aligns with concerns that current education strategies may be insufficient to support accurate and consistent HBPM in Australia [14].

Integrating HBPM into routine care also requires clear processes for interpreting and acting on results. In our workshops, participants highlighted the importance of follow-up pathways and raised considerations regarding how HBPM results might be communicated and incorporated into routine care. Although these discussions were not explored in depth, they suggested a need for clearer processes to support communication of HBPM readings and their integration into ongoing hypertension management. Establishing standardised pathways, such as automatic upload of readings to electronic records, structured follow-up appointments, and team-based care models, could improve clinician confidence, reduce uncertainty, and enhance the value of HBPM. Team-based models of hypertension management have been shown to improve BP control and are cost-effective, particularly when involving pharmacists and community health workers [32, 33].

### Strengths and limitations

This study is strengthened by its use of co-design workshops with diverse community and health professional participants across rural and regional Tasmania and provides rich insights into real-world practices and experiences. Including both groups enabled exploration of different perspectives on HBPM, highlighting tensions and opportunities for integration.

However, one key limitation is that this was a targeted analysis arising from an unexpected finding, meaning the study was not originally designed to explore these perspectives in depth. Other limitations include the focus on a single Australian state, which may limit transferability to other contexts, and the reliance on self-reported practices, which may not fully capture actual behaviour. In addition, demographic data collection was limited to age, gender, employment status, participant group, and region of participation. Characteristics such as socioeconomic status, educational attainment, healthcare utilisation, and health literacy were not collected and may influence how individuals access, understand, and use HBPM. While generalisability is not a primary aim of qualitative research, the findings may be informative for other rural and regional settings facing similar access barriers to primary care.

### Conclusion

HBPM is widely accepted by both health professionals and community members as a valuable tool for hypertension management, but current use is highly variable and often inconsistent with evidence-based protocols. Addressing barriers to high-quality HBPM will require standardised education to cover all aspects of correct HBPM, including clear systems for integration into primary care. Supporting both patients and providers with structured resources and defined feedback pathways may enhance confidence, improve BP management, and help address access-related inequities. While grounded in the Tasmanian context, these findings offer insights relevant to other rural and regional settings facing similar barriers, positioning HBPM as a particularly valuable strategy when implemented with appropriate support.

## Summary

### What is known about the topic

- Home blood pressure monitoring (HBPM) is recommended in Australian and international hypertension guidelines to improve diagnostic accuracy and support management.
- When performed according to standardised protocols, HBPM improves cardiovascular risk prediction and reduces white-coat and masked hypertension effects.
- Evidence suggests adherence to HBPM protocols is inconsistent which undermines reliability and clinician confidence.

### What this study adds

- In rural and regional Tasmania, HBPM is widely accepted and preferred by community members but commonly performed in ad hoc, non-standardised ways.
- Both health professionals and community members demonstrate limited awareness of evidence-based HBPM protocols, with variability in technique, recording, and follow-up.
- Gaps in education, communication pathways, and integration into primary care workflows reduce the clinical utility of HBPM despite strong motivation to use it.

## Data Availability

The detailed materials and methods, and data that support the findings of this study are available from the corresponding author upon reasonable request.
